# 

**Published:** 1905-09-15

**Authors:** 


Vol. LIX. SEPTEMBER 15, 1905. No. 9.

### THE DENTAL REGISTER

EDITED BY

NELVILLE S. HOFF, D.D.S.,

ANN ARBOR, MICH.

PRICE, ONE DOLLAR A YEAR IN ADVANCE.

Published Monthly by
SAMUEL A. CROCKER & CO.,
Ohio Dental and Surgical Depot,
35 to 39 W. 5th St., Cincinnati, O.

Entered at the Postoffice at Cincinnati O. as second-class mail matter

